# Non-Hodgkin Lymphoma (NHL) in Pakistan

**Published:** 2012

**Authors:** Shahid Pervez

**Affiliations:** 1 *Aga Khan University Hospital, Stadium Road, Karachi*

**Keywords:** Non-Hodgkin, lymphoma, NHL, Pakistan

## Abstract

Lymphomas are classified as Hodgkin’s and Non-Hodgkin’s lymphomas (HL; NHL); NHL being further sub-divided into B, T and Null cell categories on the basis of WHO classification. With a few exceptions worldwide, B-NHL are more common, accounting approximately 80-85% of all cases of NHL compared to T-NHL, which accounts for about 10-15% of all NHL cases. The incidence of NHL has shown a steady increase and attention is being focused on the possible causes of this increase. Epidemiologic studies indicate that environmental factors do play a role in the causation of NHL, such as drugs, pesticides, solvents, hair dyes, viruses (EBV, HTLV-1, Hepatitic C and HIV) and Helicobacter pylori. Hence many different environmental factors of risk acting on large segments of the population can contribute for increase of NHL.

Karachi reflects the global increase in the incidence of lymphoma, predominantly contributed by NHL. According to Karachi cancer registry the age standardised incidence rate of (ASIR) was 5.3/100,000 in males and 4.1/100,000 in females in 1995. A gradual increase in the annual incidence was observed during the study period, with NHL incidence increasing in 2002 to 8.4/100,000 in males and 6.5/100,000 in females, almost double the 1995 rates (1). According to Shaukat Khanum Memorial Hospital, Lahore cancer registry NHL is listed as the 4th topmost malignancy in all age-groups and both sexes combined (2). On gender basis, NHL was the 3rd most common malignancy in males while in females it stood at number six. Most of their cases were referrals from Punjab (77.56%) followed by Khyber Pakhtoonkhwa (13.60%). In other retrospective and prospective analysis in various medical institutions of Pakistan, it appears as we move from south towards north of Pakistan, incidence of NHL and its hierchy on the list of topmost cancers increases. For instance as per AFIP, Rawalpindi Pakistan cancer registry lymphomas prevalence is very high, only surpassed by prostate and skin malignancies in males (3). In children as well, lymphomas and leukemias constituted main bulk of childhood malignant tumours (4). In children as per SKMCH & RC cancer registry (1994-2010), NHL stands at number three after acute lymphoblastic leukemia (ALL) and HL (2). This most likely has something to do with various environmental factors. In our experience renal amyloidosis which does have strong association with NHL is extremely prevalent in KP. 

One thing is common however in all reports, overwhelming majority in adults being ‘Diffuse Large B-Cell Lymphoma (DLBCL)’ (5). Small B-cell NHL mostly comprised of low grade B-NHL are uncommon. In Aga Khan University histopathology archives small B-cell NHL only comprised of 8.1% of total NHL. Out of this small lymphocytic lymphoma (SLL) were 41% followed by folicular lymphoma (FL), 33% and mantle cell lymphoma (MCL) 11% (6). 

Major known risk factors like immunodeficiency or HIV does not seem to explain such a high incidence of NHL in Pakistan as fortunately incidence of HIV is still low and in overwhelming majority no such history was present. Helicobacter pylori infection though common in Pakistan, low grade MALT lymphomas of stomach associated with it are rare (7). 

Several adverse genetic alterations in DLBCL of Pakistani patients were however more prevalent including BCL2 gene rearrangements & protein overexpression as well as p53 overexpression (8). Another striking observation is that among various mature T-NHL, Anaplastic Large Cell Lymphoma (ALCL) is the most common T-NHL in our experience (9). 

Other T-NHL types like ‘Peripheral T-NHL’, ‘Angioimmunoblastic T-NHL’ are less prevalent than ALCL; however they show high association with EBV in contrast to ALCL which has very weak correlation (10, 11). 

## Conclusion

In conclusion, great majority of adult NHL in Pakistan fall into the category of intermediate to high grade with DLBCL & ALCL being the most common B & T NHL in Pakistani adults. Though we do have some clues about the interplay of various environmental and genetic factors in our population, large prospective studies to assess various environmental and genetic factors are needed to devise interventional strategies.
